# Supplementary material for: Stable skyrmion bundles at room temperature and zero magnetic field in a chiral magnet
Source: Nat Commun. 2024 Apr 22;15:3391. doi: 10.1038/s41467-024-47730-6 (PMC11035646; doi:10.1038/s41467-024-47730-6)
Supplement: Supplementary file 1 — Supplementary information [file 41467_2024_47730_MOESM1_ESM.pdf]

Supplementary information

Stable skyrmion bundles at room temperature and zero magnetic field in a chiral magnet

Yongsen Zhang[1,2#], Jin Tang[3#*], Yaodong Wu[4], Meng Shi[1,2], Xitong Xu[2], Shouguo Wang[5], Mingliang Tian[2,3] and Haifeng Du[2*]

[1]University of Science and Technology of China, Hefei 230026, China

[2]Anhui Province Key Laboratory of Low-Energy Quantum Materials and Devices, High Magnetic Field Laboratory, HFIPS, Chinese Academy of Sciences, Hefei, Anhui 230031, China

[3]School of Physics and Optoelectronic Engineering, Anhui University, Hefei, 230601, China

[4]School of Physics and Materials Engineering, Hefei Normal University, Hefei, 230601, China

[5] Anhui Key Laboratory of Magnetic Functional Materials and Devices, School of Materials Science and Engineering, Anhui University, Hefei 230601, China

[#]These authors contribute equality to this work.

*Corresponding author:

jintang@ahu.edu.cn and duhf@hmfl.ac.cn

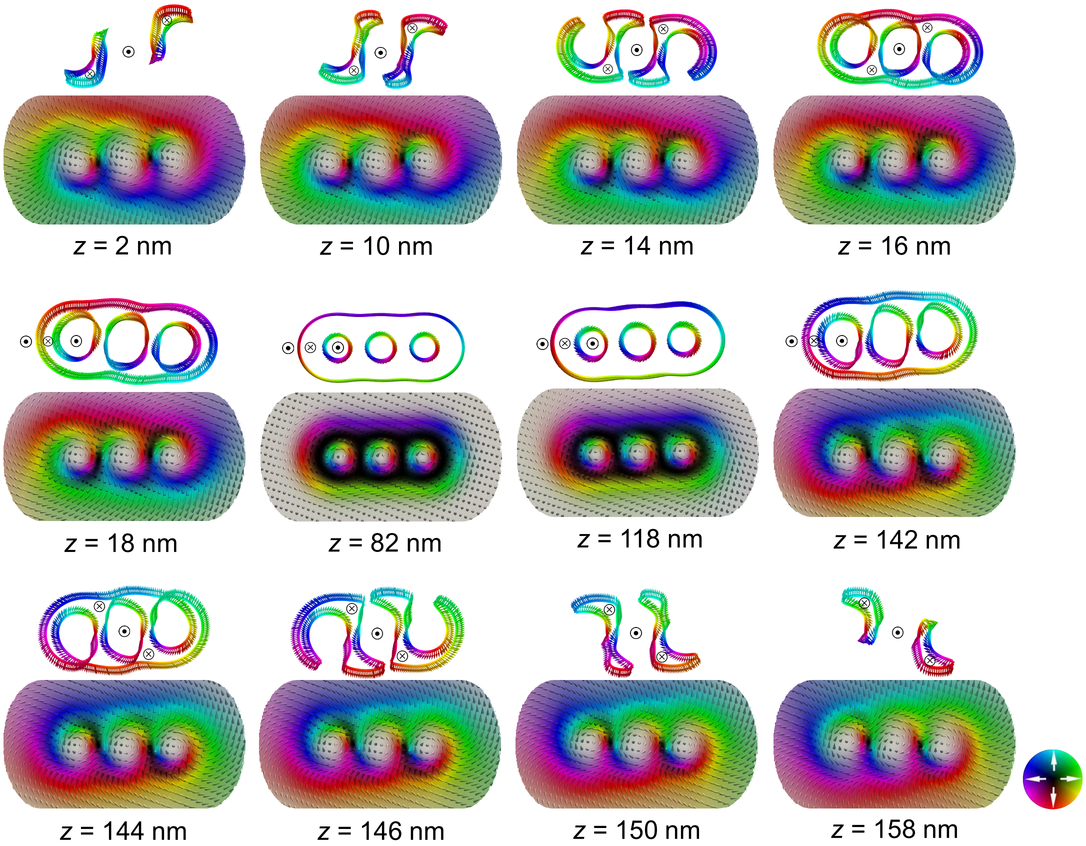

Supplementary Fig. 1 | Contour of m_z = −0.1 and magnetic configurations at different layers of the Q = 2 bundles. The colorwheel represents the in-plane magnetizations.

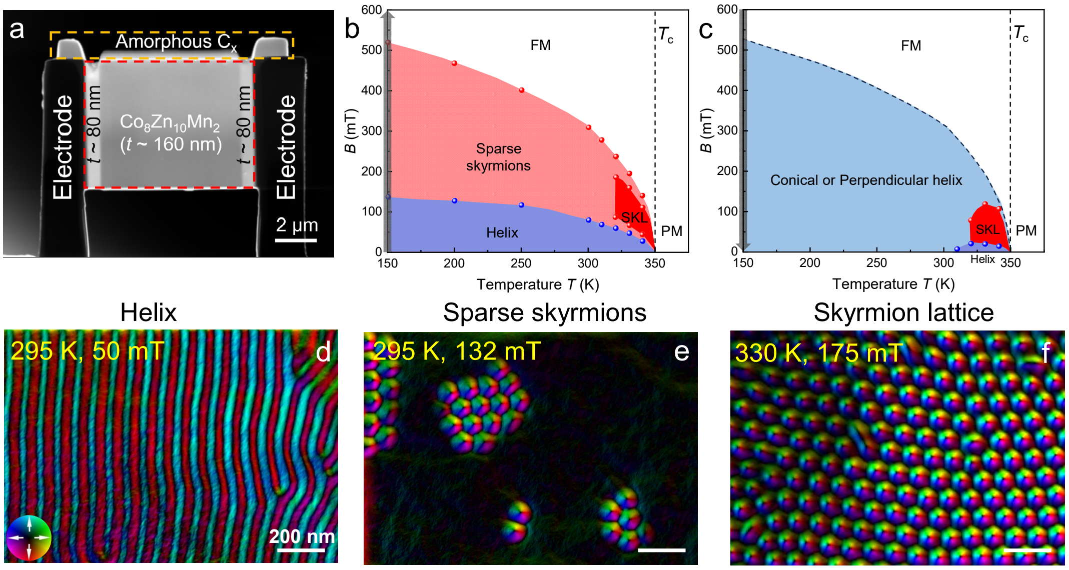

Supplementary Fig. 2 | Magnetic phase diagram of a $Co_8Zn_{10}Mn_2$ lamella. a, Overall view of the $Co_8Zn_{10}Mn_2$ microdevice obtained from scanning electron microscopy imaging. **b** and **c** Magnetic phase diagram of a 160 nm thick plate from zero-field to FM and FM to zero-field, respectively. **d-f**, In-plane magnetization mapping of the helical at $T \sim 295$ K and $B = 50$ mT, sparse skyrmions at $T \sim 295$ K and $B = 132$ mT, and skyrmion lattice at $T \sim 330$ K and $B = 175$ mT. The colorwheel represents the in-plane magnetization distributions in **d-f**.

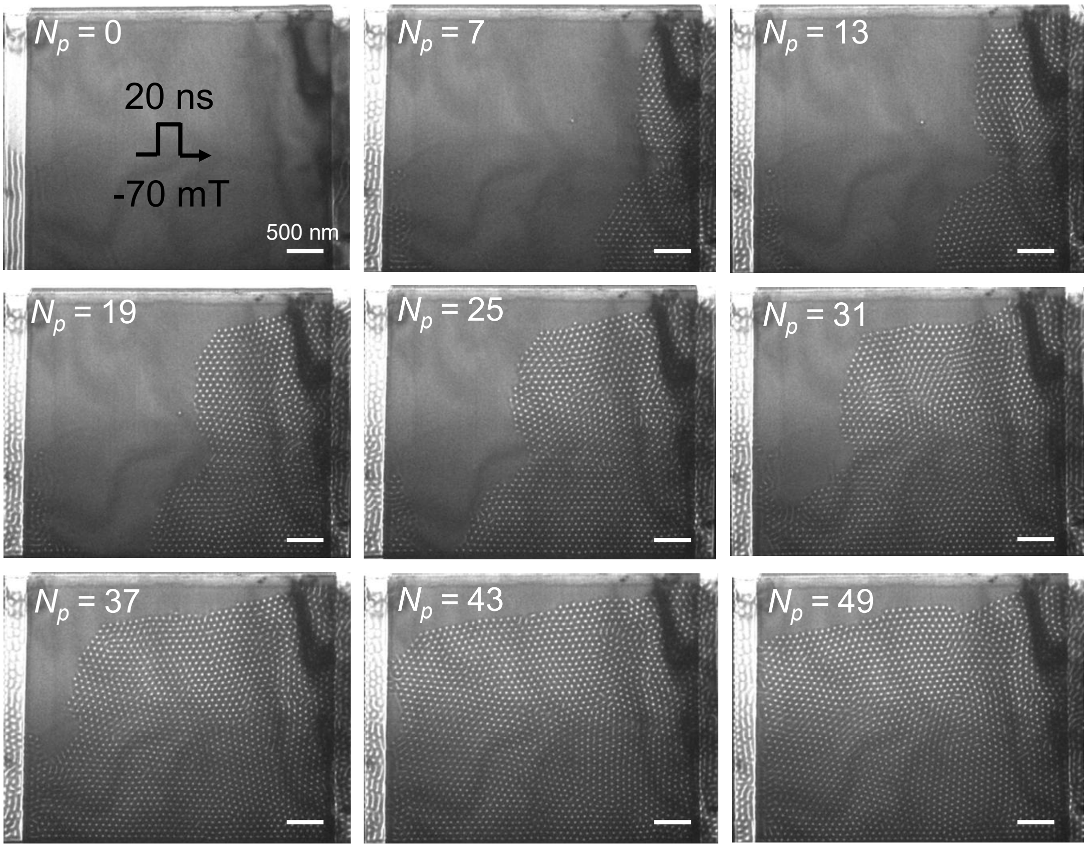

Supplementary Fig. 3 | Current induced transformation from conical to skyrmion lattice at room temperature. A sequence of Lorentz TEM images of the skyrmion creation process after applying different numbers of current pulse. Defocused distance, −1200 μm. Magnetic field −70 mT.

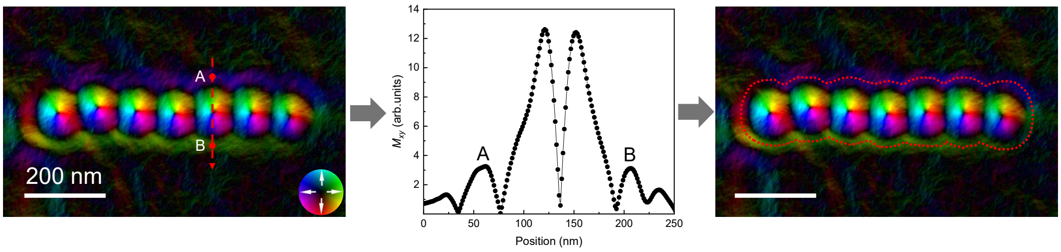

Supplementary Fig. 4 | The flowchart of skyrmion bundles area determination. The area enclosed by the red dashed line represents the region occupied by the skyrmion bundle. The colorwheel represents the in-plane magnetizations.

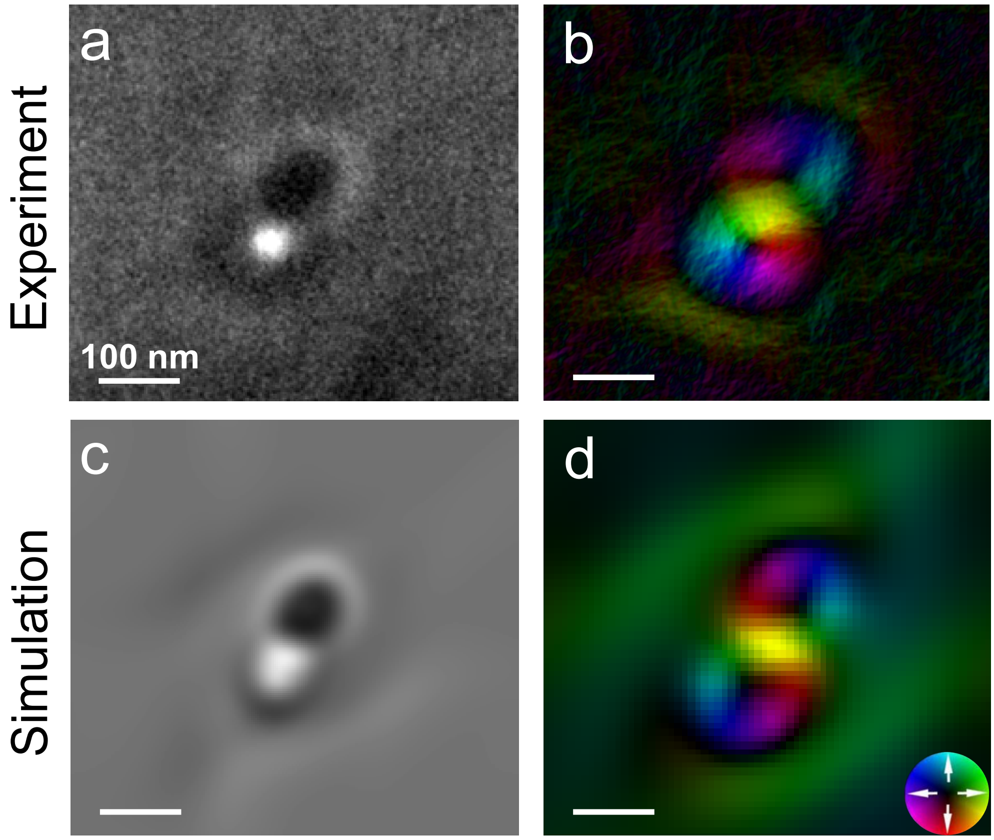

Supplementary Fig. 5 | A skyrmion-antiskyrmion pair induced by the in-plane current. **a**, Lorentz TEM image of skyrmion-antiskyrmion pair. **b**, Corresponding to the in-plane magnetic mapping marked in a. **c** and **d**, Corresponding simulated Lorentz TEM image of skyrmion-antiskyrmion and resultant TIE analysis. Magnetic field B = 52 mT at 295 K. The colorwheel represents the in-plane magnetizations. Defocused distance, −1200 μm.

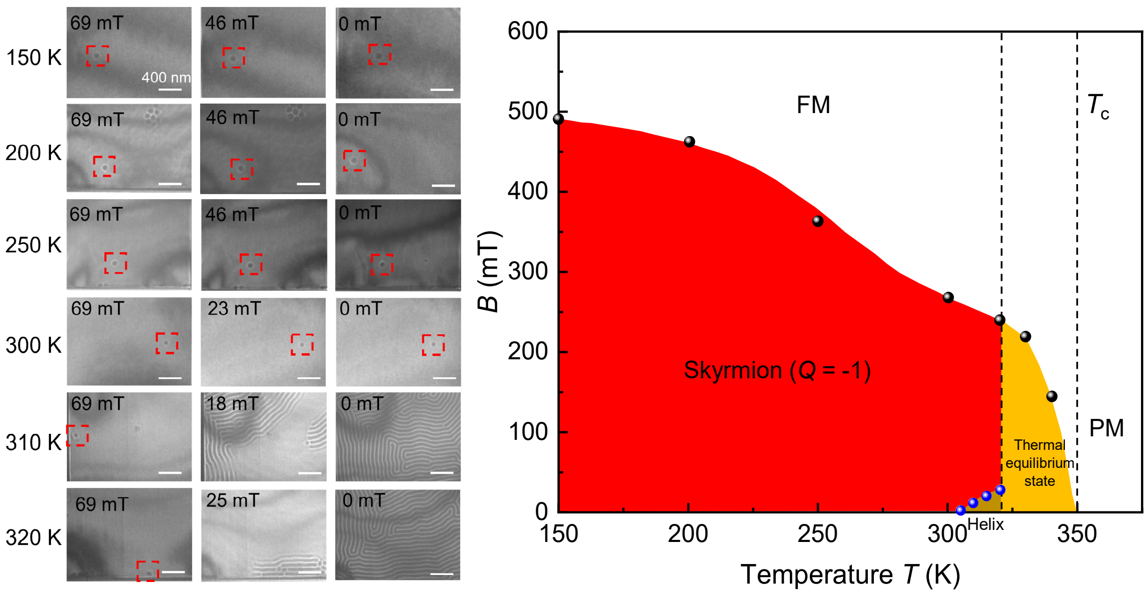

Supplementary Fig. 6 | Stability of an isolated skyrmion under zero magnetic field. The region within the red dashed-line rectangle indicate an isolated skyrmion. The data points in the diagram are the critical points of the two-state transition. FM and PM represent ferromagnetic and paramagnetic states, respectively. Defocused distance, −1200 µm.

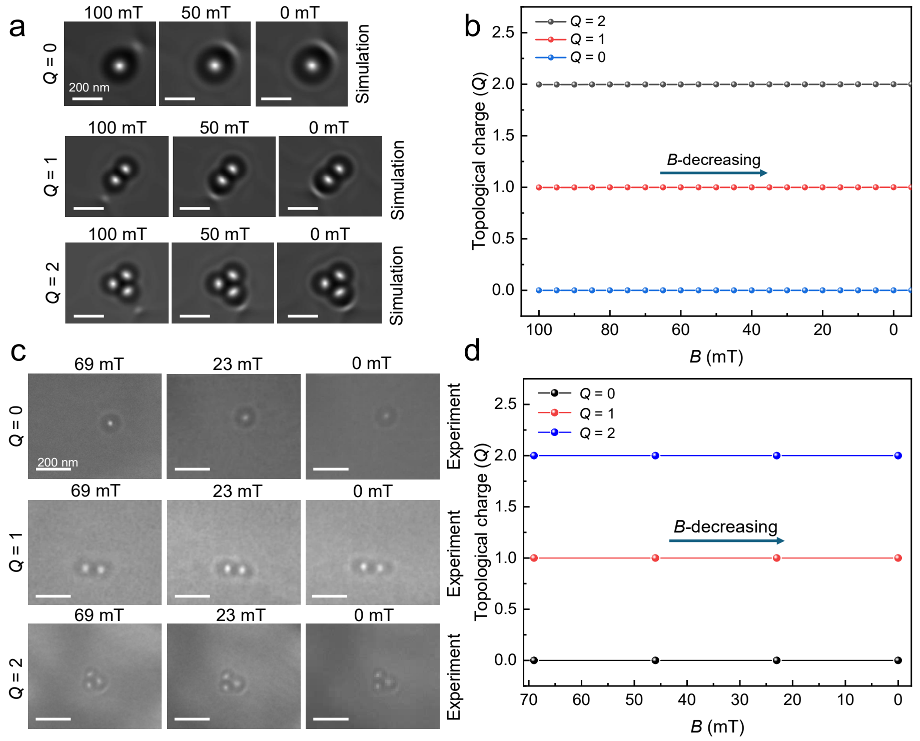

Supplementary Fig. 7 | Stability of skyrmion bundles in the field-decreasing process. a. Stability of simulated skyrmion bundles in the field-decreasing process. **b.** Corresponding simulated topological charge Q as a function of magnetic field B. **c.** Stability of experimental skyrmion bundles in the field-decreasing process. **d.** Corresponding experimental topological charge Q as a function of magnetic field B.

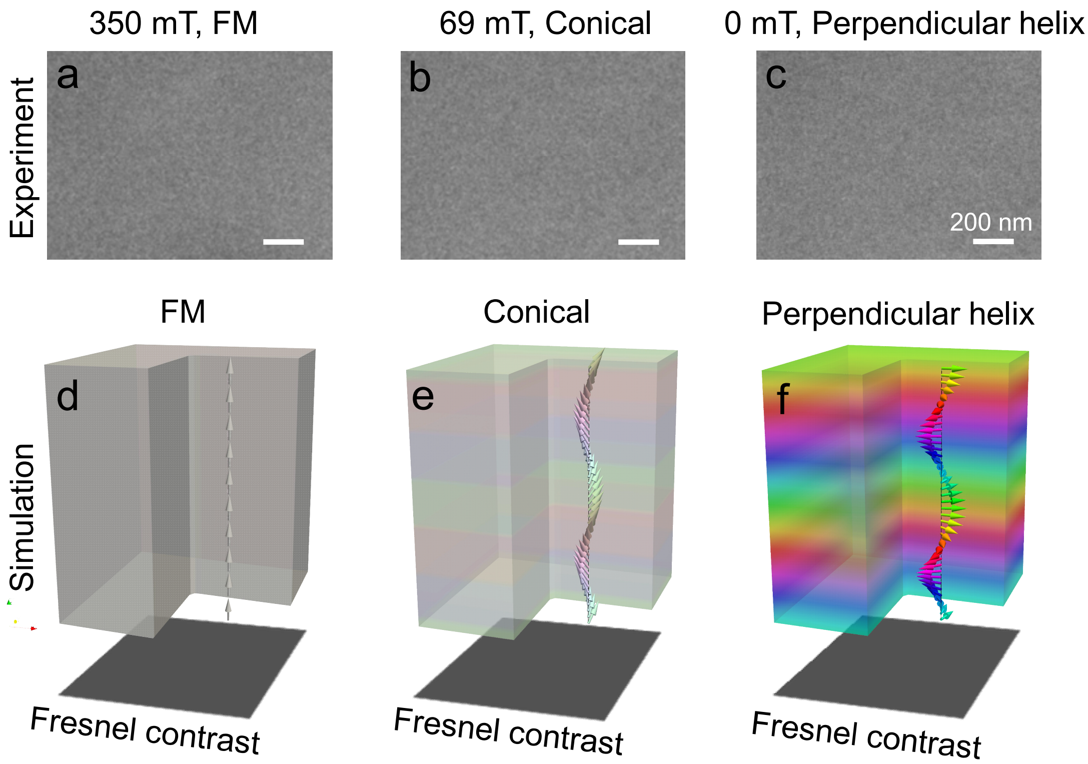

Supplementary Fig. 8 | Zero-field stability of perpendicular helix. a-c, Fresnel images from FM, to conical, and finally to perpendicular helix state as the magnetic field decreases continuously. Defocus distance, −1200 μm. **d-f,** Corresponding simulated schematic diagrams of the three-dimensional structures from FM at 350 mT, to conical at 69 mT, and finally to perpendicular helix state at zero magnetic field. The images below each display corresponding simulated Fresnel images.

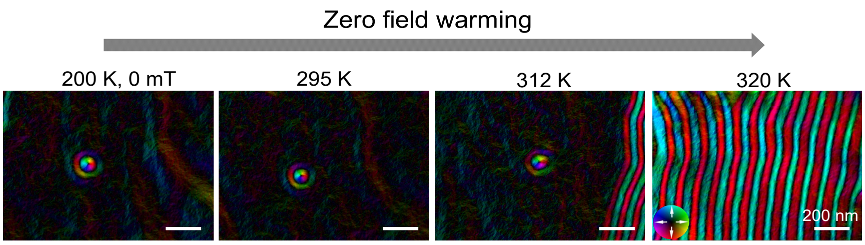

Supplementary Fig. 9 | Instability of skyrmion bundles and perpendicular helix above 320 K. The process of skyrmion bundle with Q = 0 rising temperature from 200 K to 320 K under zero-field. The colorwheel represents the in-plane magnetizations.

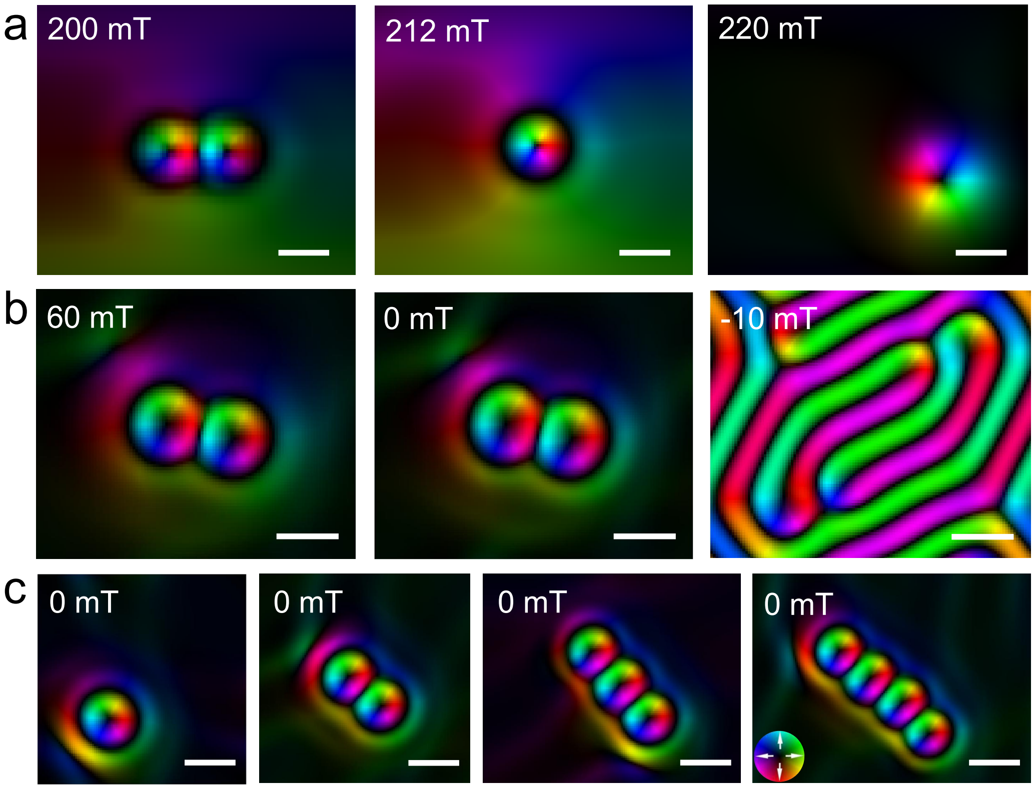

Supplementary Fig. 10 | Simulated magnetic evolutions of a Q = 1 skyrmion bundle in the varying field process. Corresponding simulated the topological quantized annihilation of a Q = 1 bundle in the field-increasing (a) and field-decreasing (b) process, respectively. **c.** Stable skyrmion bundles at zero magnetic field. The colorwheel represents the in-plane magnetizations. Scale bar, 100 nm.

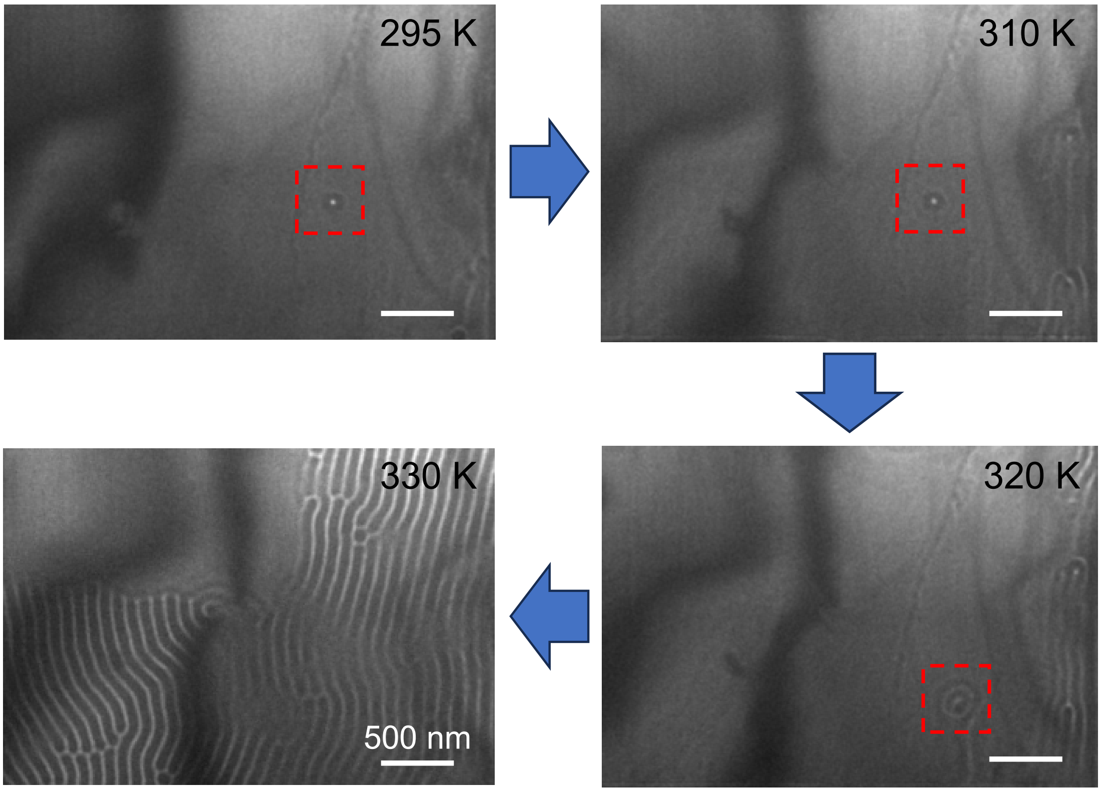

Supplementary Fig. 11 | Instability of Q = 0 bundle in a warming process. Lorentz TEM images of the Q = 0 skyrmion bundle (the red dashed-line rectangle) with the temperature increase at B = 0 mT. Defocused distance, −1200 μm.

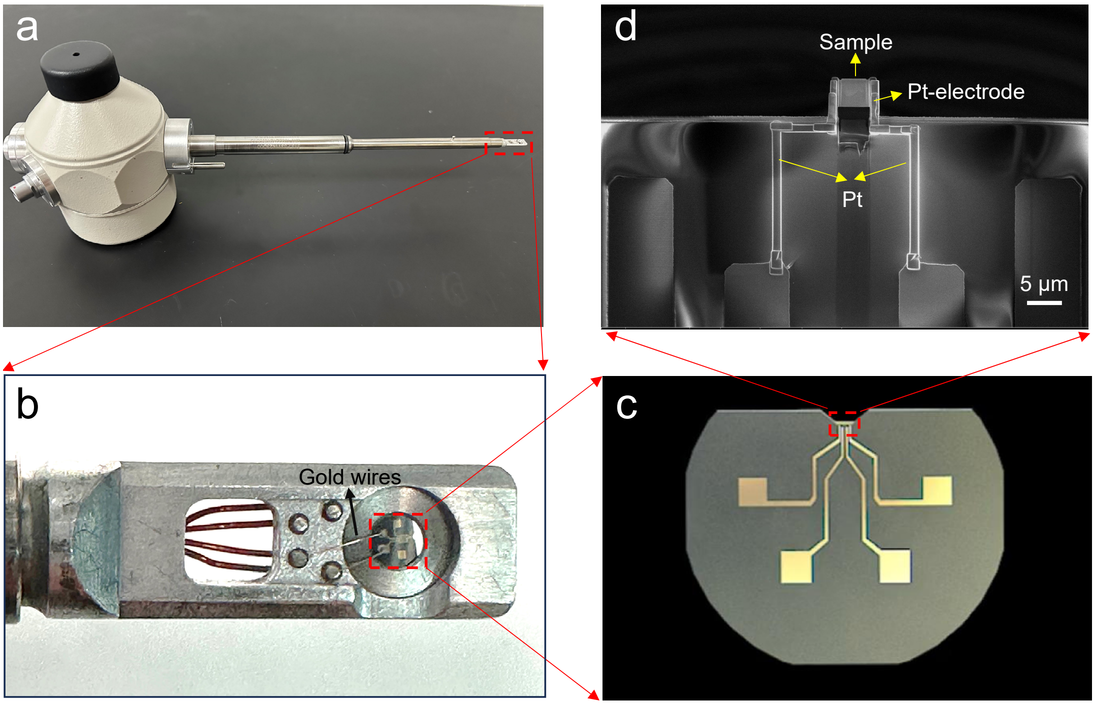

Supplementary Fig. 12 | The structure of a chip equipped with four Au electrodes and its connection to the sample holder. a, The sample holder loaded with a chip and sample. **b**, Magnified area of the red dashed line in **a**. **c**, Magnified area of the red dashed line in **b**, and displays the structure of a chip. **d**, Magnified area of the red dashed line in **c**, and illustrates the placement of the sample and the method of connection to the Au electrodes.
