# Supplementary material for: Stable skyrmion bundles at room temperature and zero magnetic field in a chiral magnet
Source: Nat Commun. 2024 Apr 22;15:3391. doi: 10.1038/s41467-024-47730-6 (PMC11035646; doi:10.1038/s41467-024-47730-6)
Supplement: Supplementary file 3 — Description of Additional Supplementary Files [file 41467_2024_47730_MOESM3_ESM.pdf]

Description of Additional Supplementary Files

File Name: Supplementary Movie 1
Description: The magneticconfigurations of the $Q = 2$ skyrmion bundlesexhibit variation along the thickness dimension.

File Name: Supplementary Movie 2
Description: Simulated generationprocess of skyrmion from thin to thick region.

File Name: Supplementary Movie 3
Description: Experimental currentinduced creation of skyrmions at 295 K and −70mT. Current density 8.19×10^{10} A/m^2 . Pulse duration 20 ns.
